# The behaviour of adult *Anopheles gambiae*, sub-Saharan Africa’s principal malaria vector, and its relevance to malaria control: a review

**DOI:** 10.1186/s12936-024-04982-3

**Published:** 2024-05-23

**Authors:** Willem Takken, Derek Charlwood, Steve W. Lindsay

**Affiliations:** 1 Laboratory of Entomology, Wageningen University & Research, PO Box 16, 6700 AA Wageningen, The Netherlands; 2 Global Health and Tropical Medicine, Instituto de Hygiene e Medicina Tropical, Lisbon, Portugal; 3 Department of Biosciences, Durham University, Durham, UK

**Keywords:** *Anopheles gambiae*, Behaviour, Hearing, Mating, Olfaction, Oviposition, Review, Taste, Vision, Malaria control, Malaria surveillance

## Abstract

**Background:**

Mosquitoes of the *Anopheles gambiae* complex are one of the major vectors of malaria in sub-Saharan Africa. Their ability to transmit this disease of major public health importance is dependent on their abundance, biting behaviour, susceptibility and their ability to survive long enough to transmit malaria parasites. A deeper understanding of this behaviour can be exploited for improving vector surveillance and malaria control.

**Findings:**

Adult mosquitoes emerge from aquatic habitats at dusk. After a 24 h teneral period, in which the cuticle hardens and the adult matures, they may disperse at random and search upwind for a mate or to feed. Mating generally takes place at dusk in swarms that form over species-specific ‘markers’. Well-nourished females may mate before blood-feeding, but the reverse is true for poorly-nourished insects. Females are monogamous and only mate once whilst males, that only feed on nectar, swarm nightly and can potentially mate up to four times. Females are able to locate hosts by following their carbon dioxide and odour gradients. When in close proximity to the host, visual cues, temperature and relative humidity are also used. Most blood-feeding occurs at night, indoors, with mosquitoes entering houses mainly through gaps between the roof and the walls. With the exception of the first feed, females are gonotrophically concordant and a blood meal gives rise to a complete egg batch. Egg development takes two or three days depending on temperature. Gravid females leave their resting sites at dusk. They are attracted by water gradients and volatile chemicals that provide a suitable aquatic habitat in which to lay their eggs.

**Conclusion:**

Whilst traditional interventions, using insecticides, target mosquitoes indoors, additional protection can be achieved using spatial repellents outdoors, attractant traps or house modifications to prevent mosquito entry. Future research on the variability of species-specific behaviour, movement of mosquitoes across the landscape, the importance of light and vision, reproductive barriers to gene flow, male mosquito behaviour and evolutionary changes in mosquito behaviour could lead to an improvement in malaria surveillance and better methods of control reducing the current over-reliance on the indoor application of insecticides.

## Background

Members of the *Anopheles gambiae* complex of mosquitoes have probably been responsible for more human deaths than any other animal (see Appendix), principally because they are exceptionally efficient transmitters of *Plasmodium falciparum*, the most lethal form of malaria [1, 2]. This mosquito complex is a highly efficient vector of disease for four main reasons: (1) they are often highly abundant, (2) frequently bite people, (3) are highly susceptible to infection and (4) are long-lived (for a mosquito) [3]. Factors such as their propensity to enter houses to feed, the host preference and behaviour after feeding are also important. *Anopheles gambiae *sensu stricto (*s.s.*)*, Anopheles coluzzii* and *Anopheles arabiensis *are the three members of the complex that, with *Anopheles funestus*, are the primary vectors of malaria in sub-Saharan Africa.

Whilst the malaria parasite has adapted to being transmitted by these mosquitoes, the insects have adapted their ecology and behaviour to exploit humans. People unwittingly create aquatic habitats for the immature stages of the mosquito around their homes and in nearby fields, and the adult female mosquito enters houses and feeds on people at night when they are least able to defend themselves. This review describes the journey of a female *An. gambiae* from its emergence to locating and feeding on a human host, before eventually laying its eggs. It illustrates how a deeper understanding of these behaviours might lead to the development of novel methods of vector surveillance and control. The review is primarily intended as an introduction to the behaviour of this important vector for students and early career scientists updating earlier reviews on the subject [4–8].

### The *Anopheles gambiae* complex

**Fig. 1:**
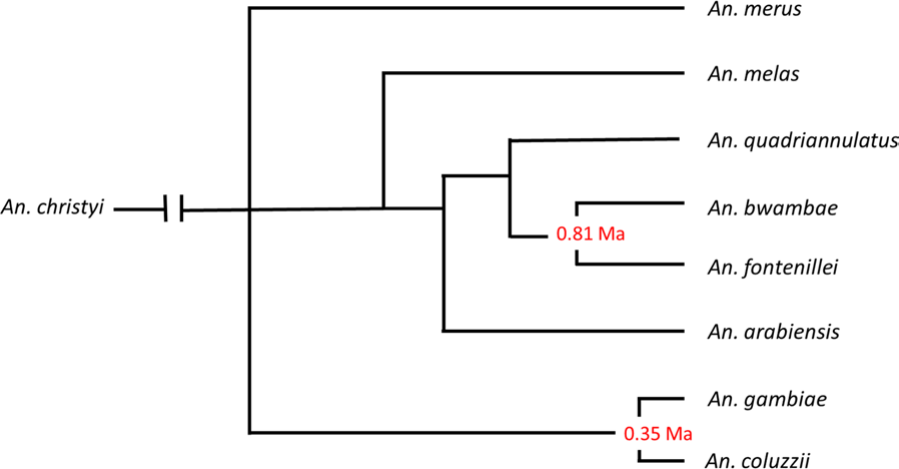
Speciation in the *An. gambiae* complex (source: Barron et al. [10]). Note that *Anopheles amharicus*, closely related to *An. quadriannulatus*, is not yet included in this figure. Numbers represent the divergence (Ma, million years ago) estimated based on the pairwise distances of the ML phylogeny and assuming a substation rate of 11 × 10^–9^ per site, per generation and 10 generations per year [38]

The *Anopheles gambiae* species complex consists of at least eight morphologically indistinguishable species, most of which are primarily zoophilic. Two of the most closely related members of the complex, *An. gambiae* and *An. coluzzii,* are primary malaria vectors due to their tendency to feed on humans, being long-lived, due to their resting inside traditional thatch roofed houses, and their relatively high abundance [9–11]. *Anopheles gambiae* occurs throughout much of sub-Saharan Africa whilst *An. coluzzii* is limited in its distribution to West Africa [12]. They are the most recently diverged members of the complex and share many behavioural traits (Fig. 1). *Anopheles coluzzii* was newly reported from East Africa for the first time suggesting that members of the complex are extending their range [13].

Until very recently humans improved the niche occupied by malaria vectors. In addition to providing a suitable larval habitat, humans provided a suitable host and so anthropophily (when blood-feeding insects prefer to feed on human blood) developed. As a side effect of anthropophily, as people improved their housing, endophily, the habit of resting inside, evolved [14]. It was these two behaviours (anthropophily and endophily) that made *An. gambiae* an exceptional vector of malaria. As pointed out by Gillies and Coetzee [15] species isolation between members of the complex ‘presumably involves a spatial element’. Spatially separated swarms, where mating takes place, is the mate-recognition system that maintains isolation between members of the *An. gambiae* complex. It is the aspect of mating behaviour that has been most thoroughly investigated, especially by Diabate and colleagues in West Africa, reviewed in Sawadago et al*.* [16].

*Anopheles arabiensis* is also a primary malaria vector in some circumstances. It has opportunistic feeding habits and will feed on both human and animal hosts, can reach high population densities but is less long lived than the other two vectors due, perhaps, to a tendency to rest outdoors [17]. It is also more drought resistant, perhaps because of its larger size, than the other two vectors and has a greater geographical distribution [18]. Due to its tendency to both feed and rest outdoors it is less susceptible to control measures, such as insecticide treated nets or indoor resudual spraying, aimed at insects which feed or rest inside houses. In a number of instances, it has replaced *An. gambiae* as the most common member of the complex when such control measures have been introduced  [17, 19, 20].

Other members of the complex (*Anopheles bwambae, Anopheles merus*, *Anopheles melas, Anopheles amharicus* and *Anopheles quadriannulatus* and, the recently described, *Anopheles fontenillei*) since they are limited in their distribution and are largely zoophilic, are only ever of minor importance as vectors.

### Life cycle of *Anopheles gambiae*

As with all mosquitoes, *An. gambiae* exploits three habitats: an aquatic environment for early development, an aerial environment where host-seeking and mating takes place and a terrestrial environment where feeding and egg production take place. Eggs are laid singly, hatch within two to three days and pass through four larval stages in seven to 10 days, depending on water temperature, food availability and quality [21, 22]. Larvae feed on organic material in the water, gently moving food into their mouths using bristles. This food is used for metabolic energy and build-up of energy reserves needed to survive the first few days as an adult mosquito [23]. Larval development, which is the only time when growth occurs, is followed by pupation that lasts two to three days [24]. In the laboratory, the period from laying to adult emergence takes 10–23 days [24, 25]. In a natural environment, daily temperature variations affect the duration of the mosquitoes’ development. Perhaps because of their slighter build, males emerge a day or two before females.

### Behaviours of *Anopheles gambiae*

#### Emergence and dispersal

**Fig. 2:**
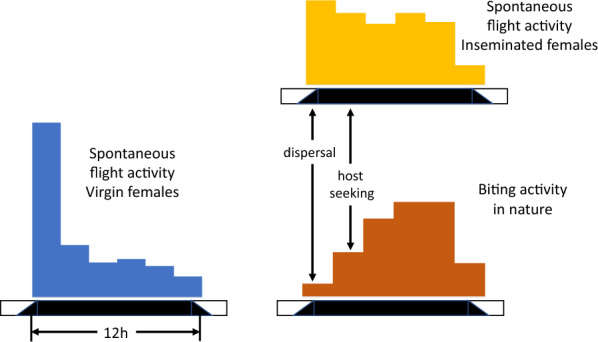
Spontaneous flight, recorded in a flight actograph, and biting activity of individual *Anopheles gambiae* after [31]. Source: [34]

Adult anophelines emerge from the pupae soon after dusk [26] and wait several minutes before inflating their wings and drying out, before flying off. The dispersal flight varies according to wind strength and direction. If there is little or no wind, dispersal can be random, but if there is a strong predominant wind, mosquitoes may be blown with the wind [27]. In open savanna, 80% of *An. gambiae* fly less than one metre above the ground [28, 29] with a maximum flight speed of 1.4–1.8 ms^−1^ [30]. Flight occurs at night and is under control of circadian rhythms [31]. Studies using flight actographs measuring the activity of individual mosquitoes show a primary peak after sunset, corresponding to the period for dispersal and oviposition, followed by secondary activity with a peak after midnight, corresponding to host location behaviour (Fig. 2).

Estimations of the distance *Anopheles* mosquitoes disperse is challenging given the heterogeneity in techniques used and the large variation in the ecology of sites [32]. Dispersal depends not only on the proximity, size and abundance of aquatic habitat to human habitation, but also how humans and alternative sources of blood meal are distributed in the landscape, the local vegetation and abiotic environmental conditions (incl. wind, relative humidity). The relative size and proximity of both aquatic habitats and human habitation is key. In the central part of The Gambia, where the river floods during the rainy season producing extensive pooling, *An. melas* may fly over 2 km from aquatic habitats to villages that are spatially clustered [33]. Support for this comes from the lack of an effect on indoor mosquito densities where large-scale larviciding was done in a 2 km area surrounding the study villages [34]. In marked contrast, in a village in western Kenya, aquatic habitats were largely human made and highly clustered within a village where houses were more widely dispersed [35, 36], suggesting short flight distances after emergence to blood feeding. When the wind is strong (> 1.2 ms^−1^), as occurs immediately preceding a tropical storm, there may be little flight activity. Passive transportation of a few individuals moving with storm fronts at high altitude has been observed [37, 38]. Whether mosquitoes that are lifted up and transported over long distances in this way survive their journey is open for debate. Even short suspension in cages at altitude reduced survival considerably [39]. Mosquitoes may also be transported long distances by planes, ships, trains and vehicles [5]. The introduction of *An. arabiensis* [40] into Brazil probably occurred by the fast mailboats from West Africa, whilst spread by vehicles was considered so important that fumigation posts were established to prevent the spread of adult mosquitoes [41].

**Fig. 3:**
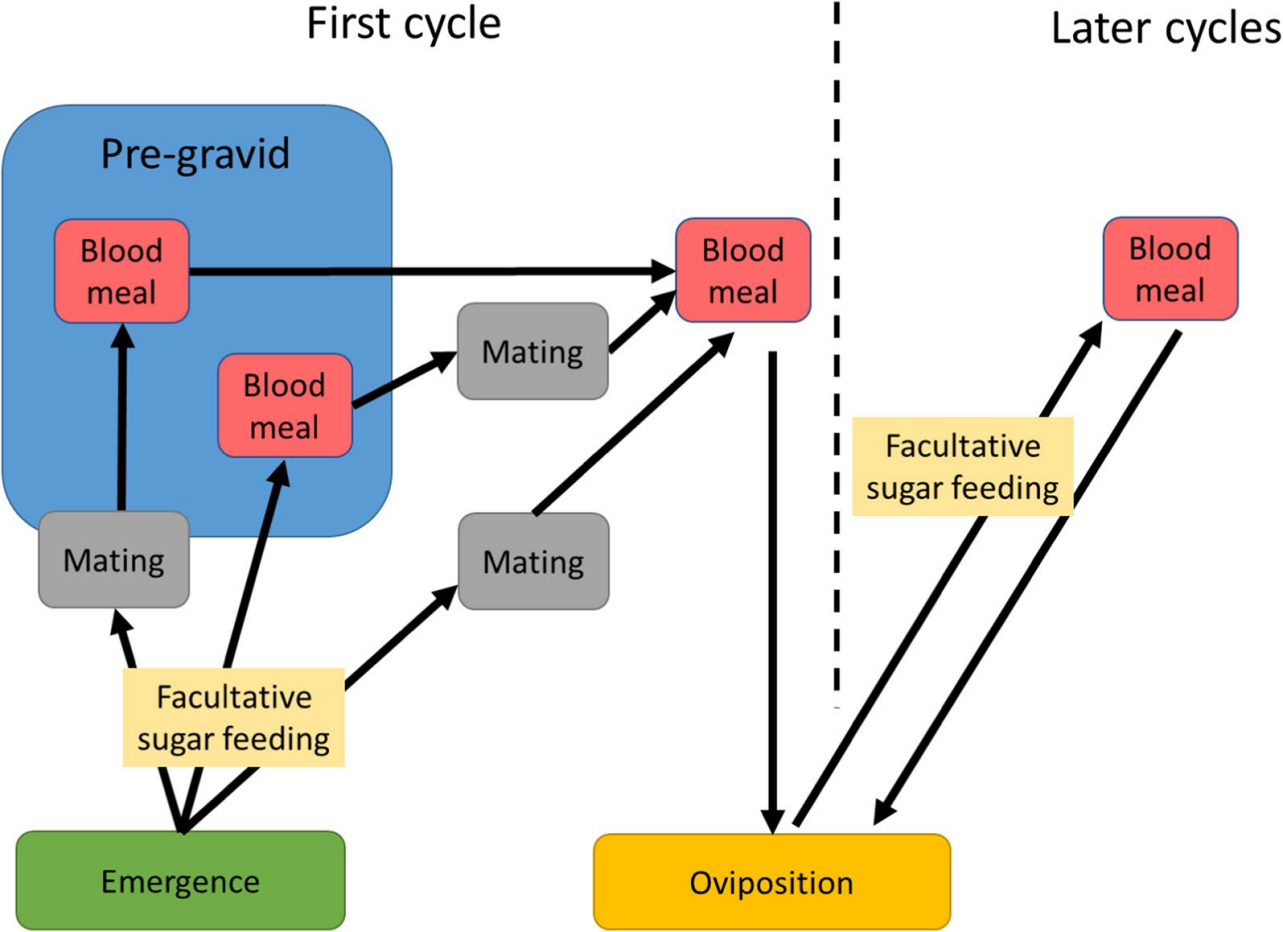
Behaviours of adult mosquitoes. Note that sugar feeding is facultative. Under natural conditions, variable proportions of recently emerged *An. gambiae* complex females may undergo a pre-gravid phase before completing their gonotrophic cycle. i.e. the period between one egg laying and the next. These females are often undernourished and require two bloodmeals in order to complete their first gonotrophic cycle [43]. A first bloodmeal may be necessary before mate-seeking behaviour takes place [44]. In the pre-gravid state (inside the blue box) females do not sugar feed as they derive sufficient nutriments from the first blood meal for mate location

After emergence, the behaviour of females of the complex is associated with three phases of adult life: mating, blood feeding and oviposition (Fig. 3). Some individuals imbibe sugar as well (see below). Newly emerged *An. gambiae* complex females may exhibit “pre-gravid” behaviour in which a proportion of the females require two blood meals before they can complete egg maturation [42, 43]. It has been suggested that the first blood meal is required to build up metabolic energy reserves when females emerge undernourished from the pupa [44] These females usually also have a smaller body size compared to their well-fed siblings and may feed before mating [43].

#### Sugar feeding

Newly-emerged adults fly to a resting site on nearby plants, such as grasses and shrubs, where they may remain until searching for a sugar meal guided by odorant volatiles from plants to which these mosquitoes are attracted [45–47]. Sugar provides a source of readily available energy whilst blood, necessary for the production of eggs, can also be used for this purpose but less efficiently [44, 48]. Sugar may be taken from honeydew of extra-floral nectaries of plants like *Mangifera indica* (mango), *Dolonix regia* (flamboyant or rural poinciana), *Thevetia neriifolia* (yellow oleanda), *Cassia siberiana* (drumstick tree), *Parthenium hysterophorus* (Santa Maria feverfew) and others [45, 49]. These sugars provide metabolic energy required for flight and mating. After a single sugar meal, females generally switch to mating followed by host-seeking and blood feeding [50]. As in all biological systems, there occurs some variation in behaviour, and recent evidence suggests that female anophelines may imbibe a sugar meal, after blood feeding [51, 52] due to the physiological condition of the female. Males continue to feed on sugar as their only source of nutrition.

#### Mating behaviour

As in other mosquito species, newly emerged male *An. gambiae* need to mature sexually before mating. During this process, their genitalia rotate 180° within a day or at most two days [53]. Females become receptive to males following a nectar feed [54, 55], or an initial blood meal, with mating taking place in swarms [55–59].

**Fig. 4:**
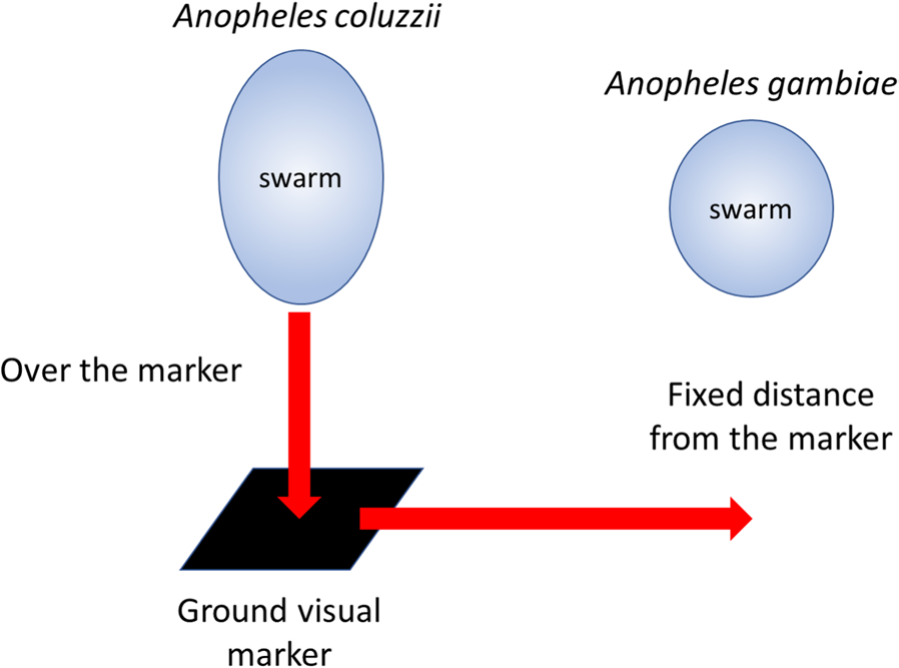
Swarming behaviour of *An. gambiae*. Note that swarms of *An. gambiae* are more spherical than *An. coluzzii* (after [60])

Swarms are characterized by the males flying in a stationary holding pattern over, or in relation to, some aspect of the environment that is species specific. Thus, *An. coluzzii* swarms directly over horizontal areas of contrast [59] whilst *An. gambiae* tends to swarm to the side of the same markers [60] ensuring some measure of spatial separation (Fig. 4). This behaviour limits hybridization in areas where the two species are sympatric. Although an individual mosquito can swarm, swarms generally contain anything from tens to 100’s of insects. The size of any swarm depends to a certain extent on the mosquito population density and the size and attractiveness of the marker [61, 62]. Virgin females respond to the same visual cues as males and in the absence of males will themselves undertake short swarming flights [56, 60]. If a swarm is present, then the female will be mated.

Swarms form for a limited time, circa 30 min, at dusk [16]. Males are attracted to the flight tone of the female which they hear using their antennae. Males beat their wings around 600 times a second (600 Hz), whereas females beat their wings at around 440 Hz [63]. Thus, the flight tones of males and females differ and males do not respond to the flight tone of other males but only to that of the female. They only hear the female when the fibrillae on their antennae are erect, which happens shortly before flight activity and which is under the control of a circadian clock [64]. As a result, receptivity to the female flight sound is limited to a relatively short period [56, 65].

Swarms can sometimes involve hundred and even thousands of individuals, hence their dynamics is particularly intriguing. It has been recently shown that the dynamics of these and other animal aggregations such as of birds, fish and insects can be recreated using relatively simple mechanistic computer models that can simulate flocking in birds, shoaling in fish and swarming in insects (Langton 1996, quoted in [66]). Thus, males and females, only need to: 1) move towards the perceived centre of mass of the insects in its neighbourhood; 2) match velocities with insects in its neighbourhood; and 3) maintain a minimum distance from other objects in the environment, including other mosquitoes. When a female enters a swarm males will follow rule #2 and match speeds (and therefore their flight tone) with the female [67, 68]. Swarm size appears not to affect mating success and it is not clear even if females select a mate [69, 70].The timing of mating is fixed throughout the year and occurs shortly before sunset. Between two closely related species, *An. gambiae* and *An. coluzzii*, there is small difference in onset of mating, which helps to keep the reproductive isolation of the two species [16].

Mating has also been reported to occur inside houses but what distinguishes this from the usual process is not known [71, 72]. Males can mate multiple times throughout adult life, but given the overall equal sex ratio, and female monogamy, they are unlikely to do so.

During mating, substances from the male accessory glands will form a mating plug inside the bursa copulatrix, which effectively blocks off the entrance to the spermatheca [73, 74]. This substance contains the steroid hormone 20-hydroxyecdysone, which induces mating-refractory behaviour in the female and stimulates oviposition [75].

#### Host location

On approaching a host, female mosquitoes may track upwind, turning into the odour stream and moving up the concentration gradient. Field experiments indicate that mosquitoes can locate host outdoors, principally carbon dioxide, from a distance of 35 m [76].

Hosts are recognized by carbon dioxide and olfactory cues (body odours mostly), emanating from the human skin. The major attracter of longer distances is carbon dioxide [76], which is a general indicator of a mammal, but closer to the host a female will be activated by a range of volatile organic compounds (VOCs) that are produced by a multitude of microbial organisms [77, 78]. Human odours include ammonia, l-lactic acid, tetradecanoic acid, 3-methyl-1-butanol and butan-1-amine [77, 79, 80]. Artificial baits, or lures, made from these VOCs, together with carbon dioxide, provide excellent ways to attract mosquitoes. These odorant cues are being recognized by selected neural receptors located on the antennae (Box 1). When stimulated by an odourant molecule, a signal will be passed through the neurons to the mosquito brain, inducing a behavioural response [81–83].

**Box 1:**
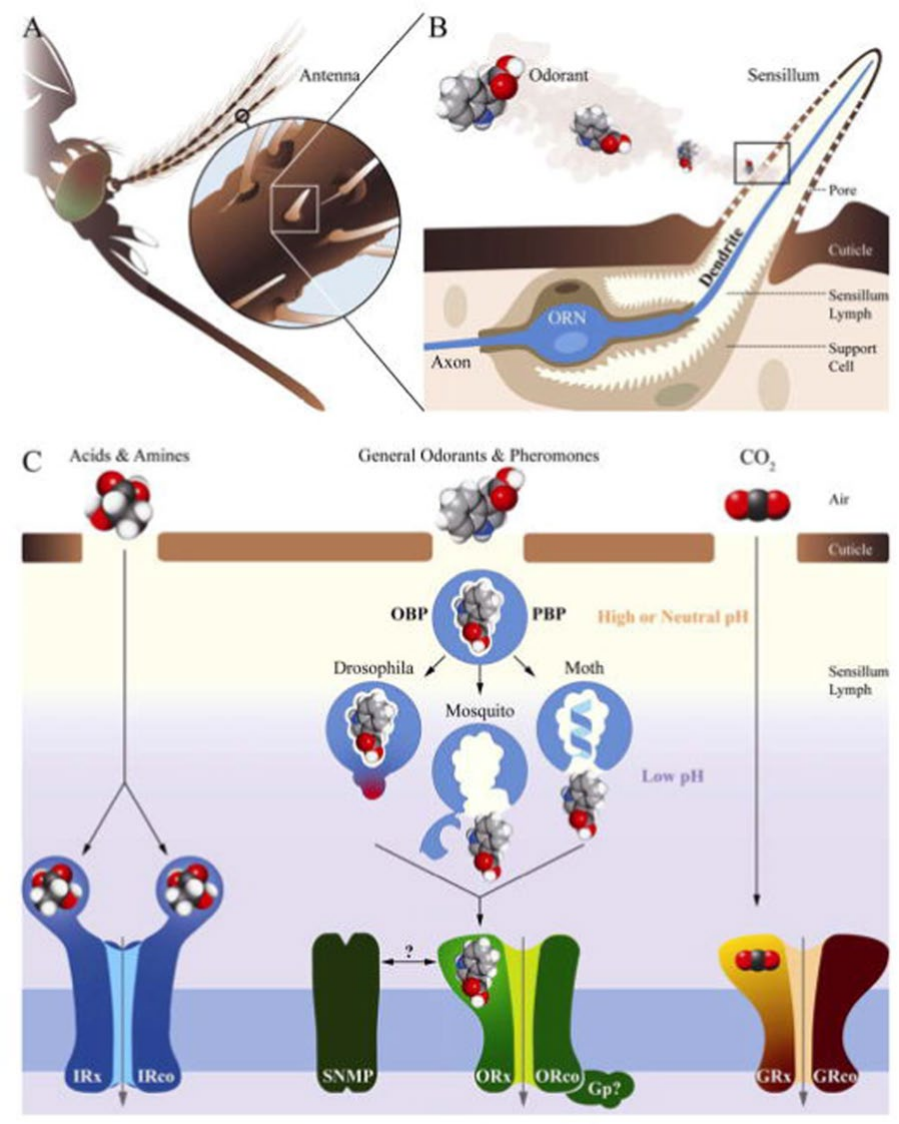
**a** An insect chemosensory system and molecular models in signal transduction. **b** An olfactory sensillum housing support cells and an olfactory neuron (blue). **c** Various groups of chemoreceptors being activated by different classes of odorants. Legend modified after [83]

Although underexplored, vision is also important for the orientation of mosquitoes. Based on studies on night-flying mosquitoes in the 1970s and 1980s [84–89], scientists showed that mosquitoes were attracted to visually conspicuous objects at a distance, although they avoided solid objects at close range. Although none of these studies involved *An. gambiae*, Gillies and Wilkes suggested that house-entering mosquitoes could be attracted to the shape of the house over 15–20 m, particularly if it was isolated from other houses or tall vegetation. Indoor lighting, visible from outside, can also attract *An. gambiae* into an experimental hut [89]. In this experiment, 84% more mosquitoes were collected in light-traps in huts with transparent walls than those with opaque walls [90]. The range of attraction of light is likely to be in the region of 5 m [90, 91], and may vary with wavelength and intensity of light. In marked contrast, light added to the Furvela tent-trap resulted in a reduced catch compared to traps without light [92] and evidence suggests that when used outdoors CDC light-traps, whilst operationally practical, do not adequately sample the outdoor biting fraction of malaria vectors [93].

As mosquitoes approach an unprotected host, temperature and relative humidity also act as attractants increasing biting rates [94, 95]. Feeding on a sitting host is primarily concentrated around the ankles and feet of individuals [96, 97].

#### House entry

**Fig. 5:**
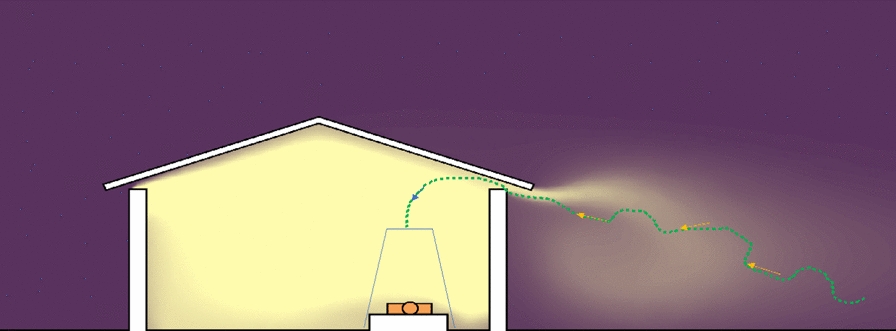
How *Anopheles gambiae* enters a house through the open eaves. Yellow represents human odours and the dashed line the trajectory of a blood-questing mosquito (After Spitzen et al. [104]). The human host is protected by a bed net

The presence of a gap between the top of the wall and the roof, or at the gable ends of a house are common features of traditional thatched-roof African houses (Fig. 5). It is through these gaps that host-seeking mosquitoes will enter [98, 99]. Recent studies show that the relative attractiveness of a house increases if there is a high concentration of carbon dioxide emanating from inside [102, 103]. As might be expected, the density of mosquitoes entering an occupied house increases with an increasing number of residents [102, 103]. Increasing ventilation, which dilutes the carbon dioxide, will have the opposite effect reducing house entry by *An. gambiae* [100, 101].

As a female *An. gambiae* approaches within several metres from a house, she will gain altitude and enter the building through the open eaves [104]. Raising a house on stilts several metres above the ground will reduce house entry of *An. gambiae*, probably due to reduced levels of human odour at ground level and lack of a wall to aid elevated flight [105]. In The Gambia, an experimental hut three metres above the ground housing two men had 84% fewer *An. gambiae* than a similar house on the ground. Nonetheless, adding netting or solid walls to an elevated hut will reduce this effect, increasing the numbers of mosquitoes entering the elevated room [106]. Similarly, in São Tomé, people living in houses built off the ground have significantly fewer *An. coluzzii* attacking them than people in houses at ground level [107].

Once inside the bedroom, an *An. gambiae* female will be attracted to hosts through volatiles produced by the occupants. Video recording shows that 75% of mosquitoes approach a human-occupied insecticide-treated bed net (ITN) from the top of the net around the torso, with a few individuals landing on the sides [108]. The behaviour of the mosquitoes was categorized into swooping, visiting, bouncing and resting. This behaviour is likely to be due to the warm odours rising from the host rising within the net as indicated using computational fluid dynamic modelling [109].

#### Variation in attractiveness to mosquitoes

Adult humans differ markedly in their relative attractiveness to *An. gambiae*, with some individuals receiving substantially more bites than others [110–112] with evidence that this variation is partly under genetic control [113]. Because of their smaller size, children are less attractive to mosquitoes than adults [110, 114], whilst pregnant women are more attractive to mosquitoes because of their larger size and more active metabolism compared to their non-pregnant sisters [115, 116]. Variation in attractiveness is partly explained by the variation in the skin microbiome, which results in differences in VOCs [78, 117, 118] Odours from human skin may be repellent or attractive [78], and thus provide an opportunity for selection of cues that could be used for prevention against mosquito bites. People with malaria are more attractive to mosquitoes, although this relationship is not clear. People infected with gametocytes appear to be more attractive to *An. gambiae* than uninfected people [119–121]. The increase in attractiveness was associated with raised concentrations of aldehydes.

After *An. gambiae* has identified a potential host, the combination of odours, skin humidity and body temperature induce a landing response followed by biting. Recent studies showed that odour, body heat and visual stimuli act synergistically in causing a landing response in *An. gambiae* mosquitoes [122]. In field studies in Burkina Faso, these stimuli combined attracted significantly more *An. gambiae* than either stimulus alone. Indeed, a thermal stimulus was required to obtain an optimal result [95, 123]. In Malawi, *An. arabiensis* was similarly attracted to a warm human decoy [123].

#### Blood feeding behaviour

When a suitable site for biting has been found, blood feeding can begin, taking up to 3 min to complete. Detailed accounts of the feeding behaviour of mosquitoes include Gordon and Lumsden [124], Robinson [125], Griffiths and Gordon [126], Christophers [127] and Ribeiro [128].

The labrum plays a central role in blood uptake, as it encloses the food channel [129]. Sensory receptor cells located at the tip of the labrum detect blood quality and even direct the labrum towards a blood vessel [130]. Once blood has been detected, on average, 3 µl of blood is ingested. During feeding, diuresis of the watery parts of blood occurs, considerably reducing excess bodyweight from the blood meal and allowing the mosquito to take flight and search for a resting site. It also allows the mosquito to cool off [131].

Mosquitoes with malaria parasite in their salivary glands will probe more frequently than uninfected mosquitoes [132, 133]. This behaviour results from destruction of parts of the salivary gland, reducing apyrase production, an important enzyme that prevents blood clotting. This behaviour has been observed with malaria-infective *An. gambiae* [134] and would partly explain multiple cases of malaria in the same house at the same time [135, 136].

#### Diel biting patterns

**Fig. 6:**
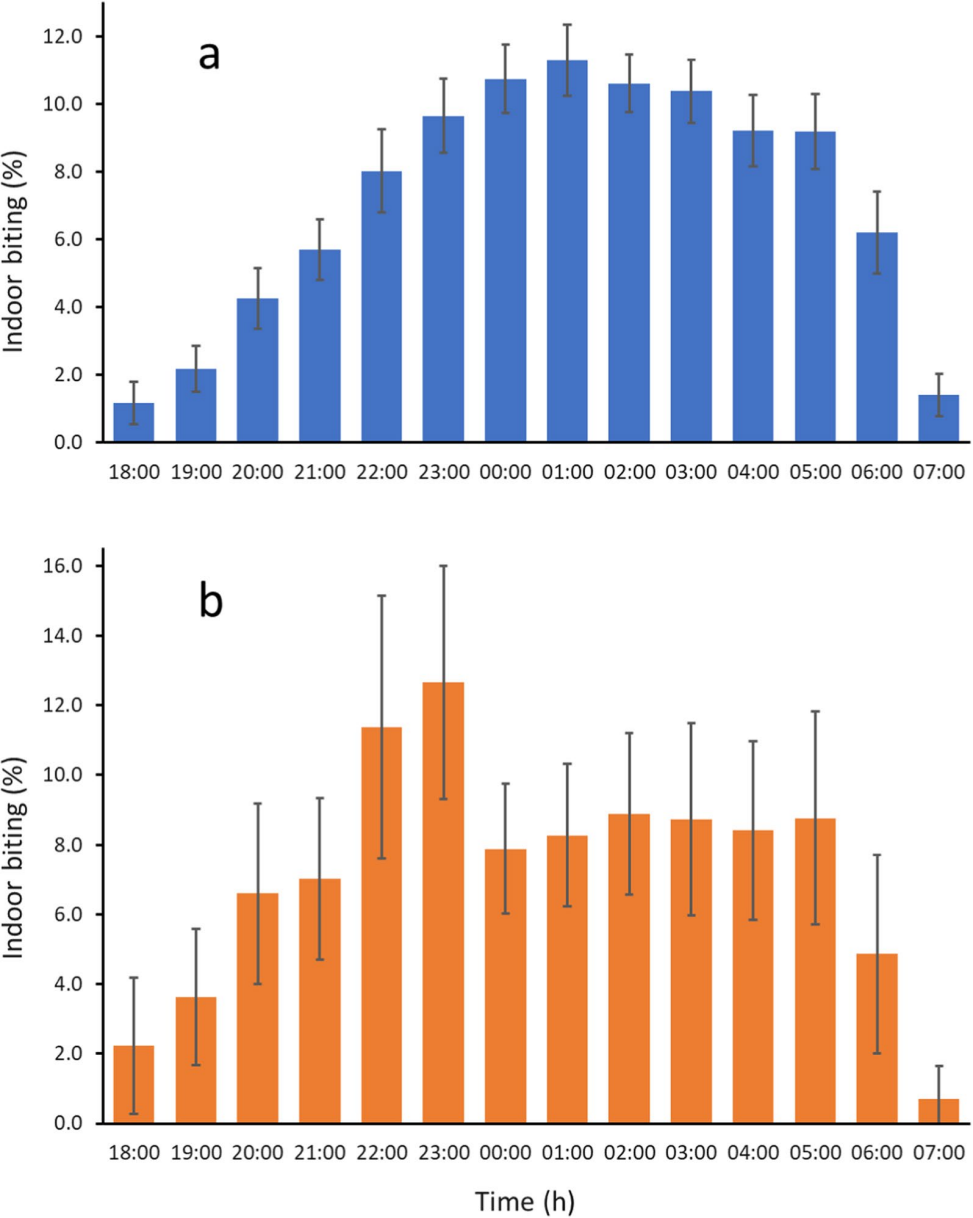
Diel biting activity of a, *An. gambiae s.l.* and b, *An. arabiensis*. Error bars are 95% confidence intervals. Data based on 92 and 17 data sets respectively. Data from Sherrard-Smith et al. [137] (data collected from 1978 to 2017)

The circadian activity rhythms (Fig. 2), distance and abundance of aquatic habitats and human population, topography, vegetation, housing quality and weather can affect the shape of the biting cycles of *An. gambiae.* This probably explains why biting patterns can vary markedly from site to site [5, 137]. Yet, summarizing 92 recent studies from sub-Saharan Africa [138] showed that there was a clear tendency for *An. gambiae* biting activity to increase from dusk to a maximum at 01.00 h before slowly declining until 05.00 h, before dropping precipitously to low levels at 07.00 h (Fig. 6).

This analysis also showed that *An. arabiensis* tends to bite earlier in the night than *An. gambiae* and *An. coluzzii* (Fig. 6b). Indeed in some cases there is marked outdoor biting early in the evening [138, 139]. In Sierra Leone young (nulliparous) *An. gambiae* bit earlier than parous ones [140]. In general, however, parous and nulliparous biting cycles are similar [5]. Mating does not appear to influence host-seeking behaviour in the *An. coluzzii* from the archipelago of São Tomé and Príncipe [141].

The paradigm of night feeding of *An. gambiae* (both *An gambiae s.s.* and *An. coluzzii*) may need to be revised, however, following the results of a recent study in the Central Africa Republic [142]. It is reported that in this study 10 to 30% of indoor bites occurs during daytime. However, this activity need not necessarily be linked to the insects circadian activity (and so is a facultative behaviour, rather than an intrinsic one). This study demonstrates that studies on *An. gambiae* feeding behaviour should be expanded to other regions to explore the nature of these behavioural changes.

#### Behavioural differences

There is a tendency to provide labels of behaviour which appear fixed, such as when mosquitoes feed on animals (zoophagy) or human hosts (anthropophagy), feed inside (endophagy) or outside houses (exophagy) or rest inside (endophily) or outside houses (exophily). This is often not the case, *An. gambiae* can be highly variable in its behaviour throughout its range. For example, if houses are well built, with few if any holes, *An. coluzzii*, that might otherwise feed on humans indoors, will feed and rest outside, on whatever hosts are available, as is the case on the archipelago of São Tomé and Principe. As pointed out by Levèvre et al*.* [143] ‘the highly anthropophilic label given to *An. gambiae s.s.* must be carefully interpreted and refer to populations rather than the whole sibling species. The same is true for endophagy and exophagy. Thus, although often considered to bite predominantly outdoors, one in three blood meals among *An. arabiensis* from Tanzania took place indoors [145]. Similarly, in a study site from Ethiopia, where people slept outdoors next to their cattle, 46% of resting *An. arabiensis* collected outdoors had fed on humans despite the high cattle: human ratio (17:1) [145]. Such results suggest that the *An. arabiensis* population was inherently anthropophagic, although this was counterbalanced by exophagic and exophilic tendencies in the mosquito [146]. Although in *An. gambiae* and *An. coluzzii* most blood feeding occurs indoors, when people are in the house and readily available as blood host, at certain times of the year blood feeding can occur outdoors, particularly when people spend the evening sitting outdoors [139]. Although *An. arabiensis* tends to feed outdoors, when hosts are scarce, they may also feed indoors [144–146]. On some occasions *An. gambiae s.s.* may feed on non-human hosts, including dogs and cattle [147], presumably as a fitness strategy. These events, however, are not common and in general humans are the preferred host for this species.

#### Indoor insecticide use

The use of insecticides for control of African malaria vectors through ITNs and indoor residual spraying (IRS) is directed at indoor-biting mosquitoes, mostly *An. gambiae*, *An. coluzzii* and *An. funestus*. It has been suggested that the extensive use of insecticides throughout sub-Saharan Africa may result in a behavioural change in the mosquitoes, leading to early-evening and outdoor biting when people are unprotected mostly outdoors where the mosquitoes are not exposed to the insecticides on interior walls or bed nets [20, 144, 148–150]. Whether such changes are due to behavioural traits selected for by the use of insecticides or simply normal variation in the behaviour of different populations needs further study. What is not debatable is that extensive use of ITNs has led to a collapse of *An. gambiae s.s.* populations in East Africa [151–153].

#### Indoor resting

After taking a blood meal, most female mosquitoes will rest indoors for two to three days while the blood meal is digested and eggs are developed. With most females, egg laying occurs after one meal, but with young mosquitoes may require two meals [44] (Fig. 3). *Anopheles gambiae*’s habit of resting indoors is a behaviour that is likely to increase the survival of a mosquito since the traditional thatched-roofed house protects mosquitoes against the extremely high temperatures experienced outdoors in the late afternoon and is an environment with relatively few predators. Metal-roofed houses, which are becoming increasingly common, are considerably hotter than thatched-roofed houses during the day and this results in decreased survival among indoor-resting mosquitoes [154, 155]. At hot times of the year, mosquitoes will move to the cooler darker and moister spaces at the bottom of the wall. Reduced mosquito survival in metal-roof houses may contribute to a decline in malaria transmission in sub-Saharan Africa.

In traditional houses, mosquitoes typically rest on the roof or base of the wall, particularly where water is stored in clay pots providing a suitable micro-climate. Blood feeding depresses mosquito activity for two to three days, and these mosquitoes are far less active than other gonotrophic stages [31, 156] resulting in most blood-feds remaining inside the house in which they fed, and only occasionally moving into neighbouring houses [157].

#### Oviposition

Eggs mature in blood-fed females within two to three days after a blood meal, after which they are ready to be laid on suitable oviposition sites. Gravid mosquitoes leave the house after dusk to find an aquatic habitat in which to oviposit. Female anophelines oviposit on still water, or on aquatic vegetation on the water surface. A median of 52 eggs are laid by individual mosquitoes, in the laboratory, and many practise skip overposition, laying their eggs in several containers [158]. *Anopheles gambiae* are generalists when it comes to selection of a site in which to lay their eggs and they are found in a wide range of habitats not just the easy-to-find hoofprint, footprint and tyre puddles. Indeed, in many places these sites are large semi-permanent or permanent water bodies in the shade or open to the sun, with clean and dirty water—although not highly odiferous water [159]. Experiments have shown that an ovipositing mosquito is attracted by water vapour [160] and by specific odours, like cedrol found in a large number of plants [161, 162] and nonane, a product from soil bacteria [163]. There are likely to be other chemical attractants and visual cues that also play a role in this vitally important part of the insect’s lifecycle. Future studies may lead to the development of novel ‘attract and kill strategies for malaria control.

#### Malaria transmission

In order for malaria transmission to occur a female *An. gambiae* needs to feed on a human that carries ‘male’ and ‘female’ (micro and macro-) *Plasmodium* gametocytes. Once in the stomach of the mosquito, the development of a flagella by the male gamete is triggered by xanthurenic acid which is a by-product of tryptophan metabolism. Exflagellation, where the eight motile gametes rupture the erythrocyte which contains them happens as a result of the drop in temperature in the mosquito. The micro-gametes move through the blood meal until they encounter a macro-gamete and fuse with it to form a motile, amoeba-like, oocyte. At this point in the life cycle the parasite is a diploid organism. At all other times it is haploid. The oocyte migrates through the midgut wall and there turns into an oocyst on the outer midgut wall. The extrinsic period of development depends critically on the environmental temperature [164]. For *Plasmodium falciparum* development of sporozoites in mosquitoes takes 11–12 days and for *Plasmodium vivax* 8–9 days at 26–27.5 °C. *Plasmodium falciparum* fails to develop at temperatures below 19 °C, whilst the lower limit for *P. vivax* is 15 °C [165]. Temperatures above 32 °C are lethal [158]. When the sporozoites are mature, oocysts rupture and sporozoites migrate through the haemocoel to the salivary glands after which the mosquito becomes infectious can pass on the parasites [166]. The percentage of female mosquitoes with sporozoites (the sporozoite rate) varies greatly, from 0 to 10% or even higher. Perhaps the factor that most affects the ability of a mosquito to be vector of disease is its survival rate. In order to become a malaria vector the mosquito has to survive through the extrinsic cycle of the parasite. This means that the mosquito, after taking an infectious meal, has to survive through four, or more, gonotrophic cycles before it will transmit.

At each phase of the gonotrophic cycle the mosquito faces different risks, each of which incurs a chance of dying. Thus, a defensive host may kill a mosquito attempting to blood feed, whilst the search for a suitable oviposition site and oviposition itself have their own risks and will depend on local conditions. For example, the risks to a mosquito that has to fly considerable distance to locate a potential oviposition site are much greater than for one where sites are to be found in close proximity to the feeding site. The risk of dying following feeding may also depend on the nature of the resting site. Mosquitoes that complete gonotrophic development inside traditional thatched roofed houses are in a more equitable environment than those that rest outdoors and so may be at less risk of desiccation. Thus, survival rate per gonotrophic cycle is important and has been estimated on a number of occasions using a variety of techniques, including dissection of the females’ ovaries to determine parous rates, mark-release-recapture experiments and estimates based on the delayed infection rate of mosquitoes [8]. In order to know the epidemiologically important figure of survival rate per day an estimate of the duration of the cycle needs to be obtained. Variations in both survival rate per cycle and cycle duration can have profound effects on the proportion of the population that might potentially become vectors. Importantly, small changes in daily survival rate can have huge consequences for the vectorial capacity of a vector [167].

On the other hand, as pointed out by [168–170], daily survival rates, determined by dissection, are remarkably similar between malaria vectors from different continents, which suggests that survival may be independent of the duration of the gonotrophic cycle. Cycle duration may, however, vary considerably with environmental factors [171].

In their meta-analysis of survival rate estimates according to methods used, Matthews, Bethel and Osei [172] obtained daily survival rates from dissections (vertical) of 0.83 (95% CI [0.80–0.86]), which was similar to the results obtained during population declines in the absence of recruitment [173]. Mark Release Recapture studies gave a lower value of 0.73 (95% CI [0.66–079]) whilst delayed infection rates (parasitological) gave 0.92, (CI [0.86–095]). Such differences result in large differences in estimates of vectorial capacity. Which of these methods provides the most accurate estimate is, however, not known. In many studies the effect of an intervention on survival is required, or estimates between different areas are needed. In such cases, for as long as similar methods are used for the estimates, absolute estimates are not needed.

### Exploiting mosquito behaviour for surveillance and control

**Fig. 7:**
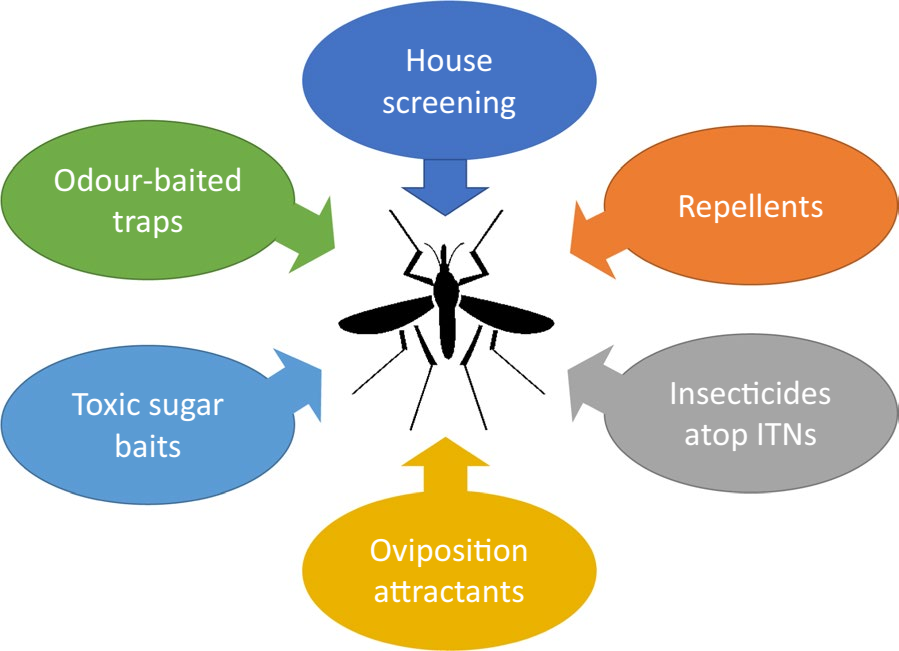
Tools for use in alternative malaria prevention or control strategies. Where ITN = insecticide-treated nets

Over the past 20 years there has been an upsurge of interest in studying the behaviour and ecology of *An. gambiae*. How has this knowledge improved the control of this medically important insect? And can this be exploited to markedly enhance the toolbox of malaria control tools [174, 175]? Here are a number of potential new tools that exploit a knowledge of mosquito behaviour and may contribute to improved malaria control (Fig. 7).

1. Screening houses
  One of the most effective methods of malaria control is prevention of parasite transmission from mosquitoes to humans by preventing the entry of mosquitoes into houses [176]. When a mosquito cannot reach or find a human host, parasite transmission is effectively interrupted, and eventually malaria will die out while uninfected mosquitoes may continue to be present as they can feed elsewhere. Interruption of transmission can be achieved by making houses mosquito proof, by closing off all possible sites of entry such as eaves, windows, doors and even cracks in the wall. In such a mosquito proof house, mosquitoes cannot enter, and hence the occupants are protected from mosquito bites and their associated *Plasmodium* infections. There is a growing body of evidence suggesting that house modification (mainly screening) is protective against malaria [177].
  Carbon dioxide is the principal cue attracting mosquitoes [76, 178], and in traditional houses carbon dioxide from human hosts is often leaking from a house through windows, doors and eaves. Understanding the movement of carbon dioxide from a house allows one to modify the design of a house to reduce carbon dixode leakage and produce a ‘stealth house’. For example, by increasing the ventilation in a house by installing three screened windows on opposite walls reduced indoor entry of *An. gambiae* by 95% [109]. Alternatively, when one makes all the walls air permeable, but not mosquito permeable, there was a 99% reduction in *An. arabiensis* compared with the comparator experimental hut with solid walls [90]. This finding is likely to results from a substantial drop in indoor carbon dioxide concentration, making it more difficult for mosquitoes to locate a host.
  Limitations: the construction of mosquito-proof houses requires the householder to pay, unlike other interventions which are given freely. Behavioural changes are needed to keep doors closed at night.

2. Repellents
  Repellents have been used as a means to prevent mosquito bites for centuries. The sensory organs of mosquitoes are activated by the repellent compound(s) leading to aversion behaviour. Historically, botanical products were used for this purpose, and since the 1950s the chemical compound DEET has been used worldwide as a skin-treatment repelling mosquitoes [179, 180]. Novel repellents, to replace DEET, are in development at a large scale, with emphasis on human safety, residual activity and ease of application. Repellents can be used in different ways with promising results as tools for malaria prevention [181–183]. Long-lasting spatial repellents offer promise in the future as they can provide an area protection against mosquitoes. Recently, promising results were reported with transfluthrin-based passive emanator designed to release transfluthrin into the air. The emanators were placed indoors [184, 185].
  Limitations: topical repellents are mostly effective as personal protection, and currently few products are available that provide community protection.

3. Incorporating highly toxic insecticide on the rooftop of ITNs
  This technology is based on the observation that, when bed nets are used, mosquitoes tend to first land on top of the net [186]. They are then exposed to an insecticide before flying off. As they spend longer on top of net than when hovering around the net, they are likely to pick up a larger dose of insecticide than when landing on the sides of the net. The discovery of increased mosquito activity at the top of the net could lead to bed nets produced with more human toxic insecticides applied to the top of the net.
  Limitations: the availability of novel effective, safe, toxicants and the additional costs that require combinations of active ingredients and a net which is more complicated to construct.

4. Oviposition attractants
  Mosquitoes ready to oviposit, find oviposition sites based on selected olfactory cues. In Kenya, the compound cedrol was found to be highly attractive to gravid *An. gambiae* [187]. In Tanzania, the compound nonane in combination with soil microbials led to significantly more *An. gambiae* eggs laid in treated water bodies than in water bodies lacking this attractant [163]. These experiments demonstrate that baiting water bodies with selective attractive cues can be used as a strategy to reduce or even eliminate *An. gambiae* populations. This can be done when the oviposition attractants are combined with a pesticide such as temephos or *Bacillus thuringiensis israeliensis* which will kill all larvae when they emerge from the eggs [188]. Studies are now needed to examine the effect of these attractants on malaria prevalence and incidence over a large area.
  Limitations: although oviposition attractants have the potential to reduce mosquito populations, studies are needed to demonstrate their feasibility. Competition with a plethora of natural sites may limit effectiveness.

5. Toxic sugar baits
  Sugar baits operate on the same principle as odour-baited traps: an attractive odour source is placed in the environment, attracting malaria vectors. By adding a toxic substance to the traps, usually a pyrethroid insecticide, mosquitoes making contact with the bait are killed. In this way, the adult mosquito population can be reduced. When critical density thresholds are reached, the malaria risk will have been significantly reduced as well [189]. A number of field trials measuring the efficacy of toxic sugar baits are currently in progress.
  Potential limitations: competition with natural sugar sources may restrict the usefulness of TSBs and there is a need for long-lasting products.

6. Odour-baited traps
  Mosquito trapping is one of the main tools used for malaria surveillance. Routine trap catches can help design and target more effective interventions. From these collections, data on species composition, nutritional state, age distribution and associated *Plasmodium* infections are obtained and, are used for estimating malaria transmission risk [190]. Transmission risk captured as the number of infective bites likely to be received during a malaria season or year, often expressed as the entomological inoculation rate (EIR), is one of the main parameters on which decisions on malaria control are based: when transmission risk is high, interventions are needed to reduce new infections and malaria incidence. This is particularly important in urban areas where it is necessary to distinguish between local transmission and imported cases.
  Odour-baited traps have been used to lower malaria transmission risk by removing the vector population with baited traps. By employing traps over a large area of several weeks, each day a fraction of the adult mosquitoes will be removed, thereby reducing the population of infectious mosquitoes continuously. Employing odour-baited traps in western Kenya, the percentage of people with malaria was reduced by more than 30% within 1 year [191]. In this study, carbon dioxide the most effective mosquito attractant, was not used. It is believed that addition of carbon dioxide or a carbon dioxide mimic may lead to strongly enhanced reduction of malaria than was achieved in this study. Odour-baited traps can also be used for mosquito surveillance, replacing the human-biting catch (HBC), which since surveillance began, has been the most-widely used trapping method [192].
  Limitations: for this tool to be effective, strong attractants need to be identified, which preferably affect more than one *Anopheles* species. Also, durable and affordable traps should be readily available, that can be distributed across large areas. The generation of large quantities of carbon dioxide will contribute to global warming.

All these interventions have the potential to select for behavioural and, where there is an active ingredient involved, physiological resistance. Behavioural resistance may occur due to avoiding an active ingredient, or in the case of house screening, exophily.

## Future behavioural research

Whilst there has been a considerable improvement in the understanding of the behaviour of *An. gambiae*, particularly over the past 20 years, there are a number of key areas of research where little, if anything, is known, some of which are highly relevant to malaria control. These include:

1. Variability of behaviour
  One of the major themes of this review is that there is considerable variation in behaviour between and within species of the *An. gambiae* complex. Yet, as far as the authors are aware, there have been no systematic reviews and meta-analyses of the behaviour of members *An. gambiae* in different parts of their range and over time. Such reviews are likely to provide important insights into the adaptability of this complex. A large number of publications on this behavioual variation have been written, and it will be interesting to explore if of concensus about a common trend can be reached. Climate change is an additional force that will affect mosquito behaviour, and development of parasites and their transmission. The huge variability in behaviour, as described in this paper, will no doubt be much affected by climate change and future studies will need to include this in understanding how this impacts *Anopheles* behaviour.
2. Movement across the landscape
  Long-distance movement of individual mosquitoes from their aquatic habitats to finding their host is poorly understood. The shape and texture of the landscape may provide natural barriers to dispersal or provide funnels to channel mosquitoes towards hosts. A deeper understanding may help us reduce malaria transmission in some areas.
3. Light and vision
  The role of visual attraction in host location is poorly understood in the field particularly for a nocturnal species like *An. gambiae*. Do mosquitoes navigate along intersections of light and dark, are they attracted to the outline of houses, and, if so does moonlight or electric lighting with tungsten or LED bulbs influence attraction or deterrence? With a rapid increase in electric lighting in parts of rural Africa it is important to understand how this may affect mosquito house entry and trapping with light traps.
4. Behavioural barriers to gene flow
  To help accelerate the deployment of gene drive mosquitoes in the field [193] it is important to understand the barriers to mating that occur in wild populations of mosquitoes and between laboratory-reared and wild mosquitoes. Whilst scientists may anticipate rapid spread of genes through a population of wild mosquitoes there are likely to be many natural behavioural barriers that restrict or prevent gene flow. For example, in a recent study in Burkina Faso, genetically-modified mosquitoes expressed reduced fitness and survival compared to wild mosquitoes [194].
5. Male mosquito behaviour
  Although understandably, most behavioural research has focused on adult females since only the female transmits malaria, there is little known bout the behaviour of adult males, apart from swarming and feeding. Although male mosquitoes use chemical cues for orientation, details about sensory behaviour of males are little known. Closer study of male behaviour may provide alternative targets for control.
6. Evolutionary changes
  Although it is well recognized that mosquitoes can adapt their behaviour in consequence of the large-scale use of insecticides [152], little consideration is given to how *An. gambiae* adapts to new environments. Africa has the fastest urban growth worldwide [195]. By 2050, the current population of 1.4 billion people is expected to increase by nearly one billion, most of these living in growing towns and cities. In some places, *An. gambiae* has already adapted to this new habitat. In the 1980s, a study in Accra, showed that *An. gambiae* had adapted to breeding in water-filled domestic containers and more polluted aquatic habitats [196]. In Dar es Salaam, a high proportion of transmission by *An. gambiae *sensu lato (*s.l*.) occurs outdoors [197]. Whether this represents a selected change in behaviour or one that results from houses with few entry points or both is uncertain. Nonetheless, it is important to detect changes in behaviour that may potentially favour malaria transmission. There is also the need to understand whether mosquitoes can adapt both behaviourally and physiological to extreme climate conditions [198].
7. Alternatives to insecticide-based interventions
  For the past 20 years, ITNs and IRS have been the main tools used for malaria control in sub-Saharan Africa. They have been remarkably successful at reducing the prevalence of malaria, but this control has stalled over the past 5–7 years and the number of malaria cases each year remains static. Increasingly, target mosquitoes develop resistance to the insecticides used, and massive research in the development of “novel” insecticides, and combination use of different insecticides, have been used to maintain the status quo of reduced *Plasmodium* parasite transmission. This race against the evolutionary strategy is likely to be lost, as it has done clearly in agriculture [199, 200]. Will the same strategy be used or the next 20 years or should one use alternative methods? With the growing urban populations, it is not feasible to deploy ITNs or IRS on a large scale due to poor compliance and some homes in informal settlements being too small to hang ITNs. Alternative forms of control need to be provided that are not reliant on synthetic insecticides, such as baited traps, push–pull systems.

## Conclusion

This review illustrates the complex series of behaviours that female *An. gambiae* use on their journey from an aquatic habitat to a blood meal, resting and oviposition. It also underlines the huge variability in behaviours seen both between species and within species underscoring the adaptability of this mosquito to different environments. Adult mosquitoes navigate these behaviours by being exquisitely tuned to environmental cues, principally semiochemicals. A century of research has illuminated our understanding of the behaviour of this important insect and has allowed us to target vector control interventions where the mosquito is most vulnerable. Many of these interventions also apply to other mosquito vector species. Basic research on mosquito behaviour underpins applied studies on vector control and it is important that funding for this research continues to be supported in the future.

## Data Availability

Data availability All data supporting the findings of this study are available from corresponding author on reasonable request.

## Appendix

### Estimating the number of malaria deaths attributed to *Anopheles gambiae s.l.*

Whilst it is impossible to make precise estimates of the number of deaths from malaria attributed to *An. gambiae*, it is likely to be considerable. It is estimated that about 241 million children under five years old have died from this disease in sub-Saharan Africa between 3000 BC and 2000 AD, making it the world’s greatest assassin. Part of the reason for this enormous attrition is because, unusually for a mosquito, *An. gambiae* evolved a propensity for feeding on people, presumably when humankind began cultivating crops, becoming relatively sedentary, with an increase in group size and sleeping in the same place for several years, making it more likely that vector populations increased in abundance increasing malaria. Shifting agriculture was the earliest form of agriculture practised in the forests of Gabon around 5000 years ago [201–203]. In this system the forest was cleared to grow crops for two to three years before moving on when soil nutrients were depleted. When the people moved to neighbouring forests, mosquitoes followed with them as humans formed a reliable food source, unlike wildlife which were relatively scarce in the rainforest with larger home ranges [203]. It is likely this would have led to an increase in malaria in early ancestral populations.

The accumulated number of malaria deaths for those aged 0–4 years old was calculated using median values of mortality determined by Snow et al*.* [204]. For the entire period pre-1960 the rate of 9.5/10,000 based on data from six studies. Was used. The population of sub-Saharan Africa was determined annually from 3000 BCE to 2000 CE by linear interpolation from estimates for specific years obtained from the literature [205, 206]. It was assumed that in 10,000 BCE that half the world’s population was living in sub-Saharan Africa, representing 3 million people. Since malaria is largely a rural disease, the rural population for Africa was estimated using data from FAOSTAT (accessed 27th April 2022) between 1950 and 2000. The 1950 estimate for 86% of the population of Africa for earlier years was used in 1950, 42.3% of the population were 0–4 years old compared to 44.3% in 2000 [207]. Annual values between 1950 and 2000 were linearly interpolated. For earlier years, the proportion of 0–4 years old were estimated from a historical study in the Kongo from 1550 to 1750 [203]. In this study the birth rate was 47 births/1000 with a crude mortality rate in children of about 25%. It is assumed that half of these deaths (12.5%) were due to causes other than malaria. Since the total African rural population was 2,580,000 (total population in Africa = 3,000,000 × 0.86 proportion living in rural areas) there would be 121,260 rural births each year with 530,512 births every 5 years (121,260) with 12.5% of these dying from causes other than malaria during this period, resulting in 397,884 survivors, ~ 15.5% of the population (i.e. 464,198/3,000,000). The assumption was that the proportion was constant from year to year before the increase in the population seen in sub-Saharan Africa from 1900 onwards. After 1900, annual values were interpolated linearly using the values given by Tabutin and Schoumaker [208].

It is estimated that 241 million children aged 0–4 years old died from malaria between 3000 BCE and 2000 CE. Making the conservative assumption that 50% of malaria deaths were due to *An. gambiae* mosquitoes feeding on people suggest that this mosquito was responsible for 120.5 M deaths. In 2021, of the 625,000 malaria deaths that occurred globally, 96% occurred in sub-Saharan Africa [209], living testament to the remarkable ability of this mosquito species, and to *An. funestus*, to transmit malaria. It should be appreciated that the estimation of deaths from malaria attributed to these mosquitoes is, understandably, an imprecise science with a wide variation in estimates. There are variations between annual population estimates by different authors [205, 210]. Estimating the proportion of children aged 0–4 years old in historical records is based on only one data source and is therefore a biased sample. Importantly, malaria deaths in older age groups or the large number of malaria deaths that would have occurred during pregnancy have been left out of these calculations.
